# THE CURRENT AND RETROSPECTIVE COGNITIVE RESERVE (2CR) SURVEY AND ITS RELATIONSHIP WITH COGNITIVE AND MOOD MEASURES

**DOI:** 10.1093/geroni/igad104.1542

**Published:** 2023-12-21

**Authors:** Stephen Aichele, Erika Borella, Paolo Ghisletta, Elena Carbone

**Affiliations:** Colorado State University, Fort Collins, Colorado, United States; University of Padova, Padova, Veneto, Italy; University of Geneva, Geneva, Geneve, Switzerland; University of Padova, Padova, Veneto, Italy

## Abstract

Cognitive reserve (CR) is often assessed with surveys spanning demographic, lifestyle, and socio-behavioral variables. The role of both past and current life experiences on CR has, however, rarely been examined. We developed the Current and Retrospective Cognitive Reserve (2CR) survey to assess classical CR proxies (socio-economic status, engagement in leisure and social activities) and other dimensions of potential importance (family engagement, religious/spiritual activity) both currently (CRc; in later adulthood) and retrospectively (CRr; as recalled from younger adulthood). We administered the 2CR, measures of general cognitive functioning, working memory (WM), vocabulary, fluid reasoning, and depressive symptoms (DS) to 235 community-dwelling Italian adults (ages 55-90 years). We used exploratory and confirmatory factor analyses to examine the 2CR latent structure, and we estimated correlations of its dimensions with cognitive abilities and DS. Analyses confirmed a three-level factor structure with two global CR factors (CRc and CRr) at the top level, dimensional CR factors (socio-economic status, family engagement, leisure activity, social engagement, and religious/spiritual activity) at mid-level, and observed items at the lowest level. Item-factor representations partially differed across CRc and CRr. Both CRc and CRr were positively correlated with measures of intelligence, WM, and DS. Associations with measures of intelligence were comparatively stronger for CRr, whereas associations of WM and DS were slightly stronger for CRc. The 2CR can be considered a reliable survey for assessing CR proxies within a multidimensional, “life stage-dependent” framework insofar as CRc are CRr closely related but also differently associated with intelligence, WM, and DS.

